# Systematic analysis of the effects of different nitrogen source and ICDH knockout on glycolate synthesis in *Escherichia coli*

**DOI:** 10.1186/s13036-019-0159-2

**Published:** 2019-04-04

**Authors:** Kangjia Zhu, Guohui Li, Ren Wei, Yin Mao, Yunying Zhao, Aiyong He, Zhonghu Bai, Yu Deng

**Affiliations:** 1 National Engineering Laboratory for Cereal Fermentation Technology (NELCF), Jiangnan University, 1800 Lihu Road, Wuxi, 214122 Jiangsu China; 2 School of Biotechnology, Jiangnan University, 1800 Lihu Rd, Wuxi, 214122 Jiangsu China; 3 Jiangsu Provincial Research Center for Bioactive Product Processing Technology, Jiangnan University, Wuxi, 214122 Jiangsu China; 4 Institute of Biochemistry, Leipzig University, Johannisallee 23, D-04103 Leipzig, Germany; 5 Jiangsu Key Laboratory for Biomass-based Energy and Enzyme Technology, Huaiyin Normal University, Huaian, 223300 China

**Keywords:** *Escherichia coli*, Glycolate, RNA-Seq, Organic nitrogen source, ICDH knockout

## Abstract

**Background:**

Glycolate is an important α-hydroxy carboxylic acid widely used in industrial and consumer applications. The production of glycolate from glucose in *Escherichia coli* is generally carried out by glycolysis and glyoxylate shunt pathways, followed by reduction to glycolate. Glycolate accumulation was significantly affected by nitrogen sources and isocitrate dehydrogenase (ICDH), which influenced carbon flux distribution between the tricarboxylic acid (TCA) cycle and the glyoxylate shunt, however, the mechanism was unclear.

**Results:**

Herein, we used RNA-Seq to explore the effects of nitrogen sources and ICDH knockout on glycolate production. The Mgly534 strain and the Mgly624 strain (with the ICDH deletion in Mgly534), displaying different phenotypes on organic nitrogen sources, were also adopted for the exploration. Though the growth of Mgly534 was improved on organic nitrogen sources, glycolate production decreased and acetate accumulated, while Mgly624 achieved a balance between cell growth and glycolate production, reaching 0.81 g glycolate/OD (2.6-fold higher than Mgly534). To further study Mgly624, the significant changed genes related to N-regulation, oxidative stress response and iron transport were analyzed. Glutamate and serine were found to increase the biomass and productivity respectively. Meanwhile, overexpressing the arginine transport gene *argT* accelerated the cell growth rate and increased the biomass. Further, the presence of Fe^2+^ also speeded up the cells growth and compensated for the lack of reducing equivalents.

**Conclusion:**

Our studies identified that ICDH knockout strain was more suitable for glycolate production. RNA-Seq provided a better understanding of the ICDH knockout on cellular physiology and glycolate production.

**Electronic supplementary material:**

The online version of this article (10.1186/s13036-019-0159-2) contains supplementary material, which is available to authorized users.

## Background

Glycolate is the smallest alpha-hydroxy acid containing both alcohol carboxy groups. It is used as a tanning, peeling, and cleaning agent in the cosmetic and textile industries [1]. Glycolate can be polymerized into polyglycolide (PGA), which has perfect gas-barrier properties and mechanical strength, making it an ideal packaging material [2].

To date, a few microorganisms have been used as host strains for the production of glycolate, including the yeasts *Saccharomyces cerevisiae* and *Kluyveromyces lactis* that were resistant to low pH [3]. Chemolithotrophic iron- and sulfur-oxidizing bacteria also produce glycolate as an exudate during the oxidation of pyrite or elemental sulfur [4]. Deng et al. reported that by overexpressing native *aceAK* and *ycdW*, and deleting competitive pathways, the engineered *Escherichia coli* strain produced more than 65 g/L glycolate, the highest glycolate titer reported to date for *E. coli* [5]. However, when the metabolic pathways of the strain were altered artificially, there were many concomitant changes, such as energy production, cofactors and demand for different nitrogen sources, remained under-investigated. Metabolomics, transcriptomics, and proteomics approaches can detect changes in intracellular metabolism, and thereby help to make product synthesis pathways more efficient. Meanwhile, RNA-Seq has revolutionized transcriptome analysis by facilitating the expression profiling of thousands of genes at the same time [6, 7]. This technique is mainly applied to study transcriptome differences caused by various treatments [8]. Therefore, through RNA-Seq we could investigate global transcriptional changes and have new insights into glycolate-producing strains [9–11].

**Fig. 1:**
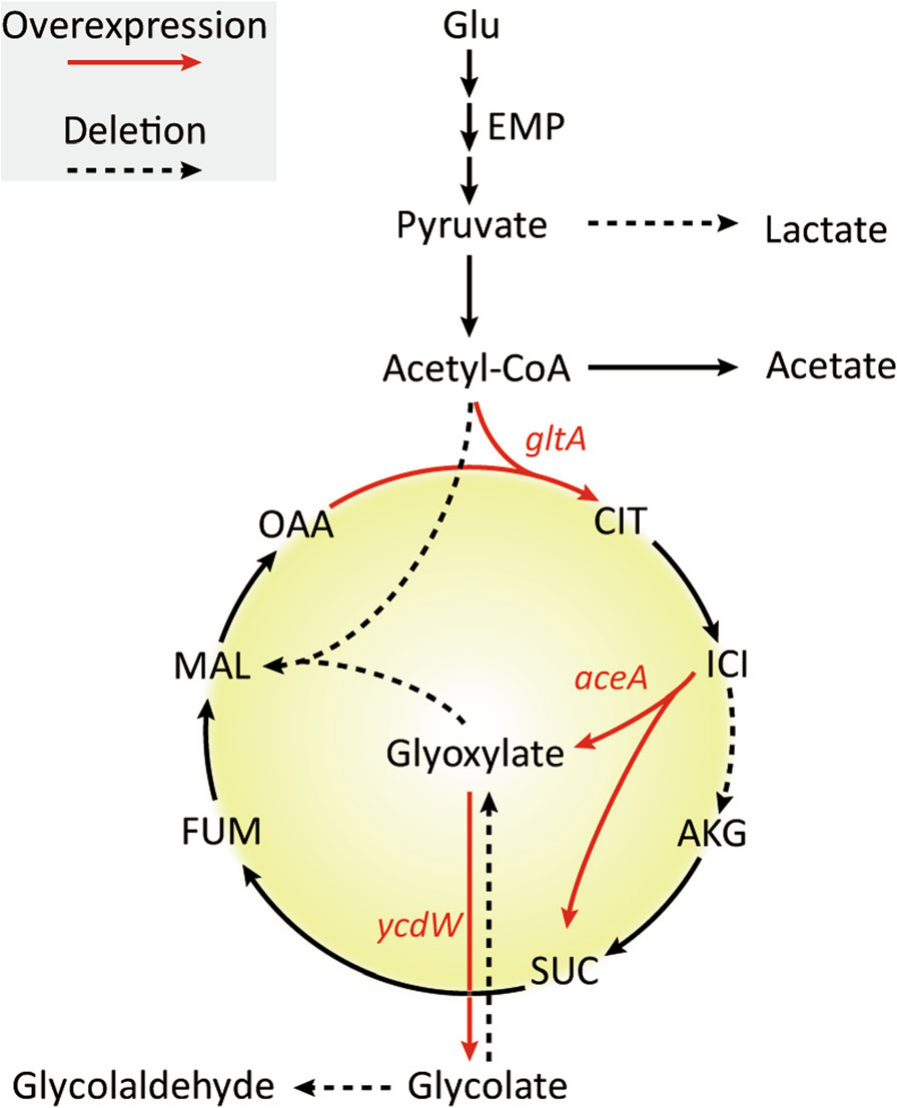
Metabolic strategies for redirecting carbon flux to glycolate in Mgly624. EMP, glycolysis pathway; CIT, citrate; ICI, isocitrate; AKG, α-ketoglutarate; SUC, succinate; FUM, fumarate; MAL, malate; OAA, oxaloacetate; *gltA*, citrate synthase; *aceA*, isocitrate lyase; *ycdW*, glyoxylate reductase

In this research, we investigated the impacts of different nitrogen sources on glycolate production in the engineered *E. coli* Mgly534 strain (Table 1) using RNA-Seq. The Mgly624 strain (Table 1; Fig. 1), which had a different carbon flux distribution between the TCA cycle and the glyoxylate shunt, was also subjected to transcriptome analysis and the transcriptional levels of genes in glycolate production pathway were combined to explain the changes in fermentation characteristics. Due to the fundamental importance of NADPH in glycolate production, the status of intracellular NADPH/NADP^+^ under different conditions and the cause of ratio changes were both investigated. In addition, the significantly changed genes in ICDH-knockout strain were analyzed, including N-regulation, oxidative stress and iron transport, to have a better understanding of Mgly624 for the further glycolate synthesis.

**Table 1:** Strains and plasmids used in this study

Name	Relevant genotype	Reference
Strains		
MG1655(DE3)	*fhuA2*	[51]
Mgly5	MG1655(DE3)Δ*ldhA*Δ*glcB*Δ*aceB*Δ*aldA*Δ*glcDEF*	[5]
Mgly6	MG1655(DE3)Δ*ldhA*Δ*glcB*Δ*aceB*Δ*aldA*Δ*glcDEF*Δ*icd*	This study
Mgly534	Mgly5 carrying pTrc99A-*aceAK*-*ycdW* and pCDF-*gltA*	[5]
Mgly624	Mgly6 carrying pTrc99A-*aceA*-*ycdW* and pCDF-*gltA*	This study
Mgly6241	Mgly6 carrying pTrc99A-*aceA*-*ycdW*, pCDF-*gltA* and pRSF-*argT*	This study
Mgly6242	Mgly6 carrying pTrc99A-*aceA*-*ycdW*, pCDF-*gltA* and pCOLA-*argT*	This study
Plasmids		
pKD4	oriR6Kγ, Kan^R^, rgnB (Ter)	[52]
pKD46	araBp-gam-bet-exo, bla (Amp^R^), repA101 (ts), ori101	[52]
pCP20	Amp^R^, Cm^R^, FLP recombinance oriR6Kγ, Kan^R^, rgnB (Ter)	[53]
pTrc99A-*aceA*-*ycdW*	Amp^R^, pTrc99A harboring *aceA* and *ycdW* from *E. coli* MG1655	This study
pTrc99A-*aceAK*-*ycdW*	Amp^R^, pTrc99A harboring *aceA-aceK* and *ycdW* from *E. coli* MG1655	[5]
pCDF-*gltA*	Strep^R^, pCDFDuet-1 harboring *gltA* from *E. coli* MG1655	[5]
pRSF-*argT*	Kan^R^, pRSFDuet-1 harboring *argT* from *E. coli* MG1655	This study
pCOLA-*argT*	Kan^R^, pCOLADuet-1 harboring *argT* from *E. coli* MG1655	This study

## Results

### Effects of organic nitrogen sources and ICDH knockout on cell growth and glycolate production

**Fig. 2:**
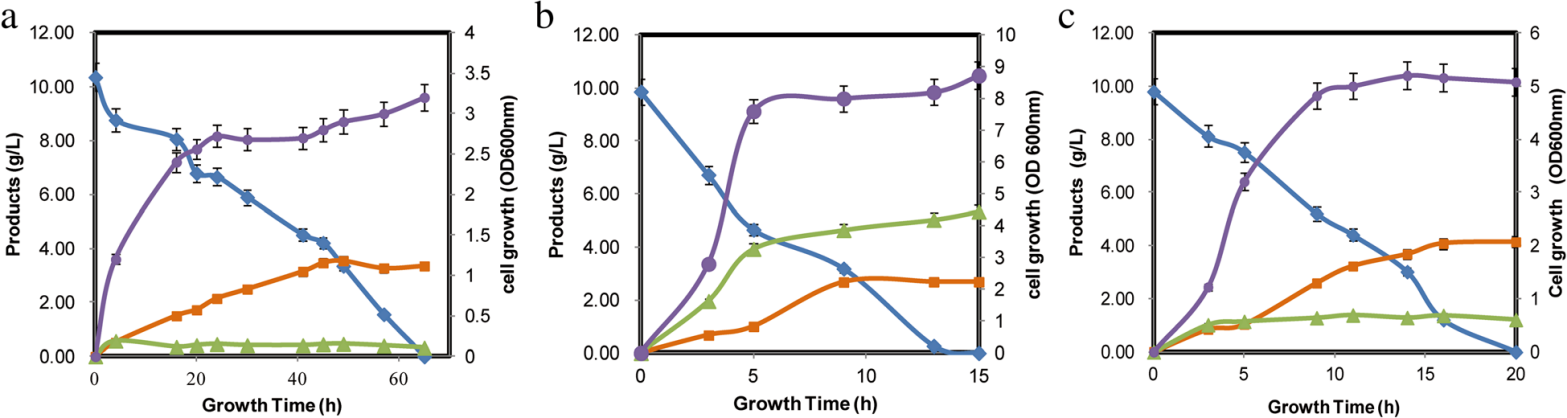
Batch fermentation process in a 5 L bioreactor. OD600nm (purple lines), glucose concentration (blue lines), glycolate concentration (orange lines), acetate concentration (green lines) are shown. **a** Strain Mgly534 cultured on inorganic nitrogen sources; **b** Strain Mgly534 cultured on organic nitrogen sources; **c** Strain Mgly624 cultures on organic nitrogen sources

When Mgly534 grew on M9 minimal medium, 24 h was needed to reach stationary phase, producing 3.55 g/L glycolate from 10 g/L glucose after 50 h (Fig. 2a). However, the slow growth and low productivity were not suitable for industry. When 2 g/L yeast extract and 8 g/L tryptone were added, it took only 8 h to reach stationary phase, and the maximum optical density (OD_600_) was increased from 3.2 to 8.7 (Fig. 2b). However, glycolate was decreased from 3.55 g/L to 2.67 g/L, while acetate was increased from 0.46 g/L to 5.33 g/L. The organic nitrogen sources accelerated cell growth but reduced glycolate production. Although Mgly534 overexpressing *aceAK* and *ycdW* achieved a high titer of glycolate, we hypothesized that disruption of the TCA cycle by deleting the ICDH might direct more carbon to the glyoxylate shunt for glycolate production. Thus, the ICDH in Mgly534 was deleted to generate strain Mgly624, which could not grow on M9. With the addition of 2 g/L yeast extract and 8 g/L tryptone, which were best for cell growth and glycolate production in Mgly624 (Additional file 1: Figure S1), 4.14 g/L glycolate was produced in the 5 L bioreactor (Fig. 2c). Compared with Mgly534, Mgly624 produced more glycolate, with 0.81 g glycolate/OD (2.6-fold higher than Mgly534). To explain the effects of organic nitrogen sources and ICDH knockout on glycolate production, the transcriptome analysis was performed on these three fermentation conditions.

### Global analysis of metabolism in the engineered strains by RNA-Seq

In the analysis of transcriptome samples, ‘A’ represents the fermentation of Mgly534 on M9 medium with NH_4_Cl as nitrogen source and ‘B’ represents the fermentation of Mgly534 in M9 medium with additional tryptone and yeast extract as nitrogen sources. ‘C’ represents the fermentation of Mgly624 in M9 medium with additional tryptone and yeast extract as nitrogen sources. Cells from the 5 L bioreactor were harvested at exponential phase, and three samples (each with two independent biological replicates) were sequenced on an Illumina Hiseq sequencer, generating average of 4 Gb per sample. All samples had Q20 values > 96% and Q30 values > 90.5% [12]. More than 90% of reads could be mapped to the *E. coli* K-12 MG1655 genome (NCBI accession no. NC_000913.2). We introduced fragments per kilobase of transcript sequence per millions base pairs sequenced (FPKM) value to facilitate the comparison of different transcription levels between samples. FPKMs for every gene were calculated and are available at Additional file 2. Thus, 377 novel transcripts were identified among all samples.

The criteria for categorizing significantly differential expression of genes (DEGs) were 2-fold up- or down-regulation (|Log_2_ Fold Change > 1|) with a false discovery rate (FDR) < 0.05. Compared to ‘A’, expression of 99 genes in ‘B’ was decreased and 185 genes were elevated. Compared to ‘B’, 19 genes in ‘C’ were down-regulated and 49 genes were up-regulated.

Gene Ontology (GO) enrichment was performed to identify the putative functions of DEGs in each group [13], and the results are shown in Additional file 1: Figure S2a,b. The majority of DEGs in the two groups were related to catalytic activity, binding, cell part, and metabolic process subcategories.

**Fig. 3:**
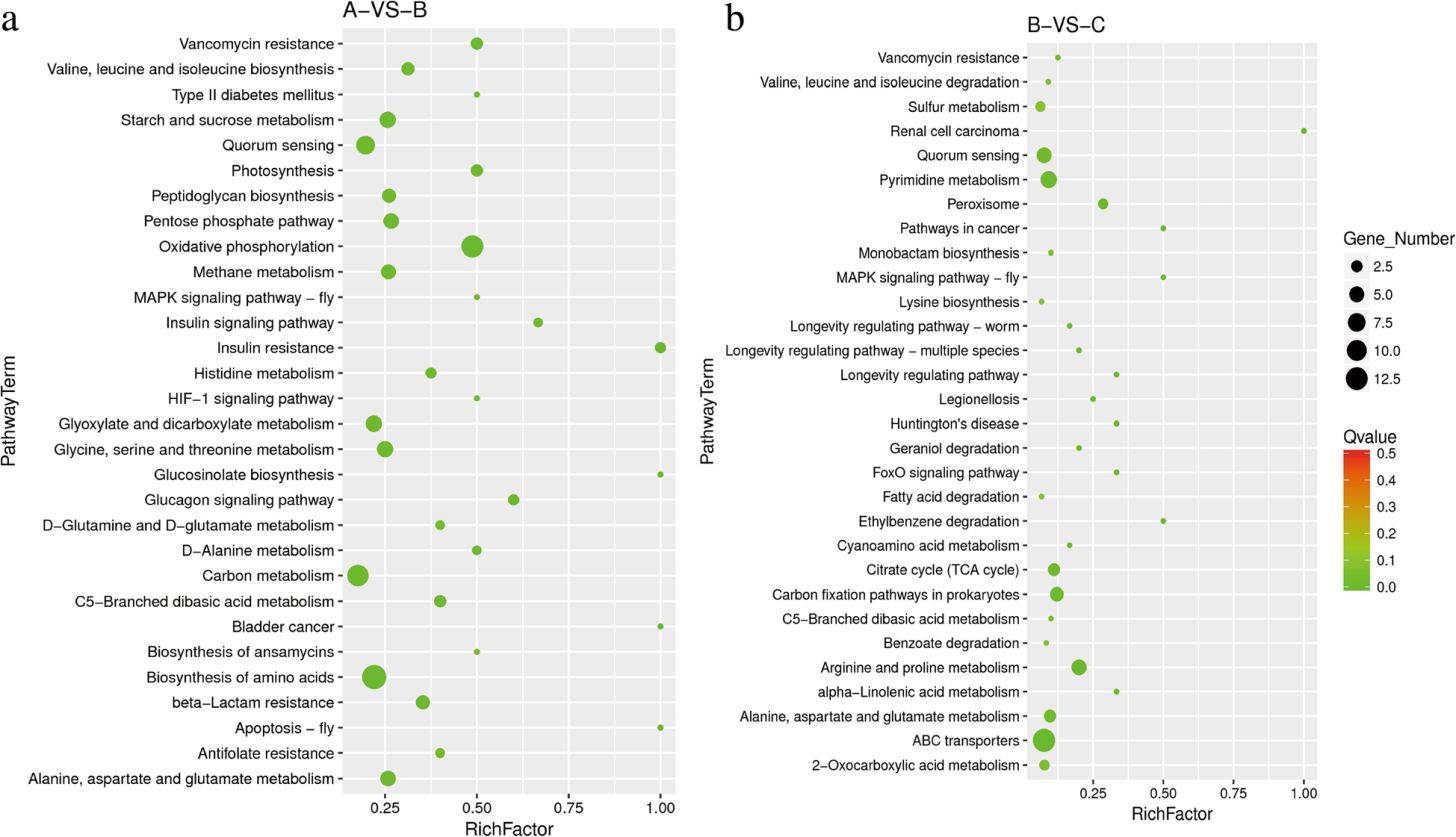
KEGG enrichment scatter plot of differentially expressed genes (DEGs) in Group A vs. B (**a**) and Group B vs. C (**b**). The vertical axis represents the pathway names, and the horizontal axis represents the rich factor. The size of the point indicates the number of DEGs in the pathway, and the colors of point correspond to different Q-value ranges

The results of Kyoto Encyclopedia of Genes and Genomes (KEGG) enrichment analysis of A vs. B are shown in Fig. 3a. When organic nitrogen sources were present, cell growth and carbon metabolism were significantly increased. The most significantly up-regulated DEGs were linked to oxidative phosphorylation, carbon metabolism, and biosynthesis of amino acids. The results of KEGG enrichment analysis of B vs. C are shown in Fig. 3b. In ‘C’, synthesis of α-ketoglutarate, and precursors of many amino acids were severely diminished. It was obvious that the most significantly up-regulated DEGs were associated with pyrimidine metabolism, arginine and proline metabolism, alanine, aspartate and glutamate metabolism, and ABC transporters. However, the ICDH knockout resulted in other changes, such as sulfur metabolism, carbon fixation pathways in prokaryotes and peroxisome.

### The transcriptional changes directly related to glycolate production

**Fig. 4:**
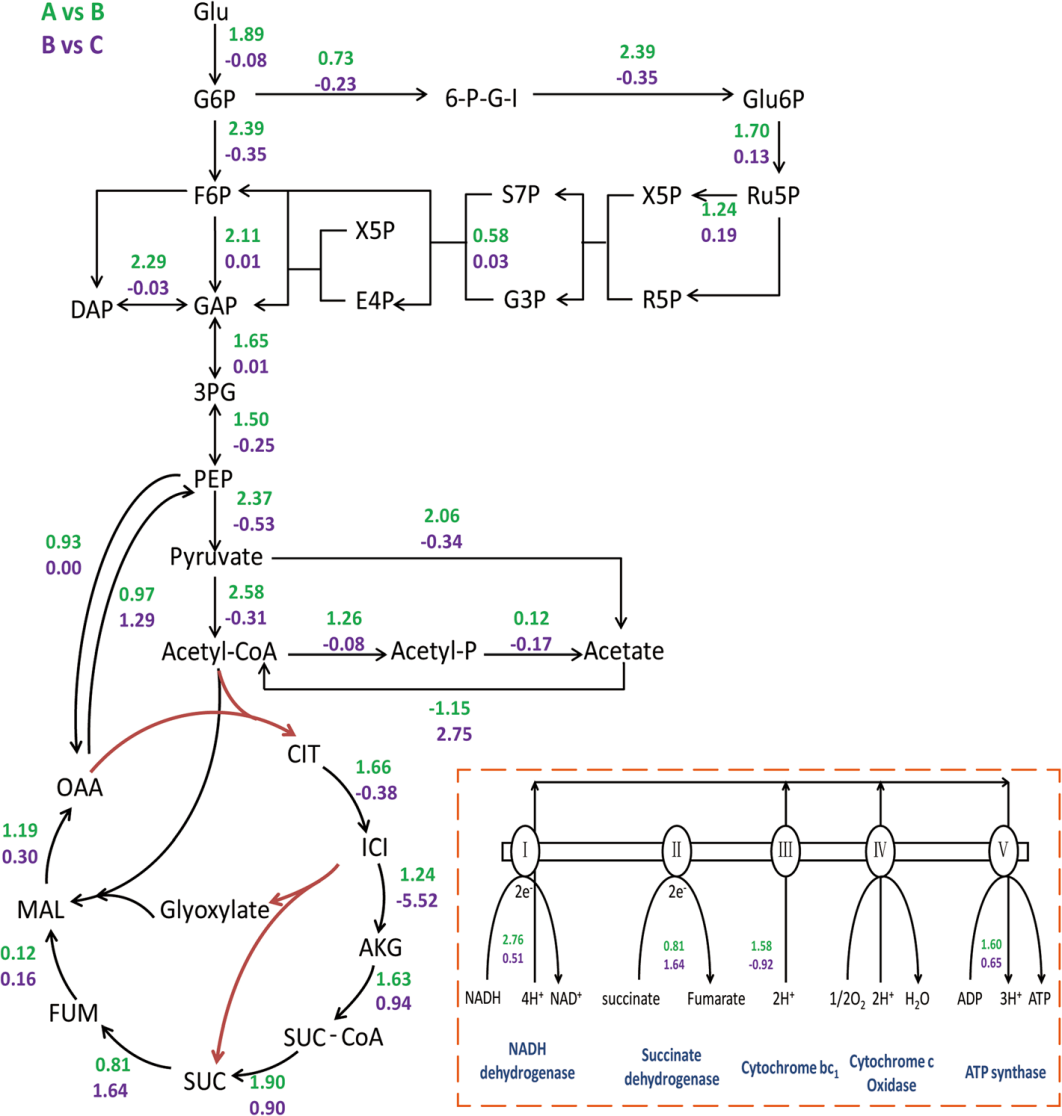
Summary of genes involved in the glycolate production pathway. Values represent the Log_2_FoldChange of genes in Group A vs. B (green) and B vs. C (purple) comparisons. Red line represents the over-expressed pathway

The synthesis of glycolate was related to the glycolysis pathway (EMP), the TCA, and the glyoxylate shunt (GS), and the Log_2_FC values of each gene in A vs. B and B vs. C comparisons were labeled (Fig. 4).

In the A vs. B comparison, all genes in the EMP pathway were up-regulated significantly. The transcription of glucose 6-phosphate dehydrogenase (*zwf*) was also up-regulated, indicating that the pentose phosphate (PP) pathway could be increased to support cell growth with higher NADPH. In addition, phosphate acetyltransferase (*pta*) and pyruvate oxidase (*poxB*) in the acetate synthesis pathway were up-regulated by 2.39- and 4.17-fold in ‘B’. Organic nitrogen sources provided more proteins and amino acids for biomass synthesis, and the carbon in the yeast extract and tryptone could also be absorbed and enter TCA cycle. It showed that the excessive carbon from glycolysis was not needed by TCA cycle, leading to the overflow of acetate via *pta* and *poxB* [14]. Further, the presence of organic nitrogen sources changed energy metabolism in cells (Fig. 4). The transcriptional level of the respiratory chain genes in ‘B’ were significantly enhanced, accompanied by an increase in ATP production. The supply of ATP in ‘B’ could increase cell growth rate and biomass, as well as the production of recombinant protein [15].

In the B vs. C comparison, the transcription of acetyl-CoA synthetase (*acs*) was up-regulated by 6.73-fold. We hypothesized that this might reflect greater amino acid consumption in ‘C’. Consumption of some amino acids (serine and aspartate) in the medium supports cell growth and the formation of acetate, and expression of *acs* was induced to utilize acetate [16]. In ‘C’, serine transport (*sdaC*) and aspartate ABC transporter (*gltJKL*) were up-regulated by 2.64-fold and 3.12-fold respectively, which could be attributed to greater serine and aspartate consumption in ‘C’ compared to ‘B’, and so the transcription of *acs* was up-regulated, resulting to the more utilization of acetate. Compared with B, the PEP carboxykinase gene (*pck*) was up-regulated by 2.45-fold in ‘C’. In *E. coli*, PEP carboxylase catalyzed anaplerotic reaction, converting 3 carbon metabolites to replenish the metabolic pools of the TCA cycle. The reaction catalyzed by PCK in *E. coli* served to keep the relative balance between OAA and PEP pools and recycle excess carbon in TCA cycle to supply PEP for cellular requirements [17]. In ICDH knockout strain, more amino acids were absorbed into TCA cycle, for example, aspartate aminotransferase, which catalyzed aspartate to OAA, was up-regulated by 2.19-fold. As a consequence, OAA pool was higher than PEP pool, and then the activity of PCK was increased in order to recycle OAA to PEP. Although the glycolytic rate was slightly decreased in ‘C’, the recycle of acetate and oxaloacetate was improved, leading to an increase in the carbon pool for glycolate production.

### Intracellular NADPH/NADP^+^ ratio analysis

**Fig. 5:**
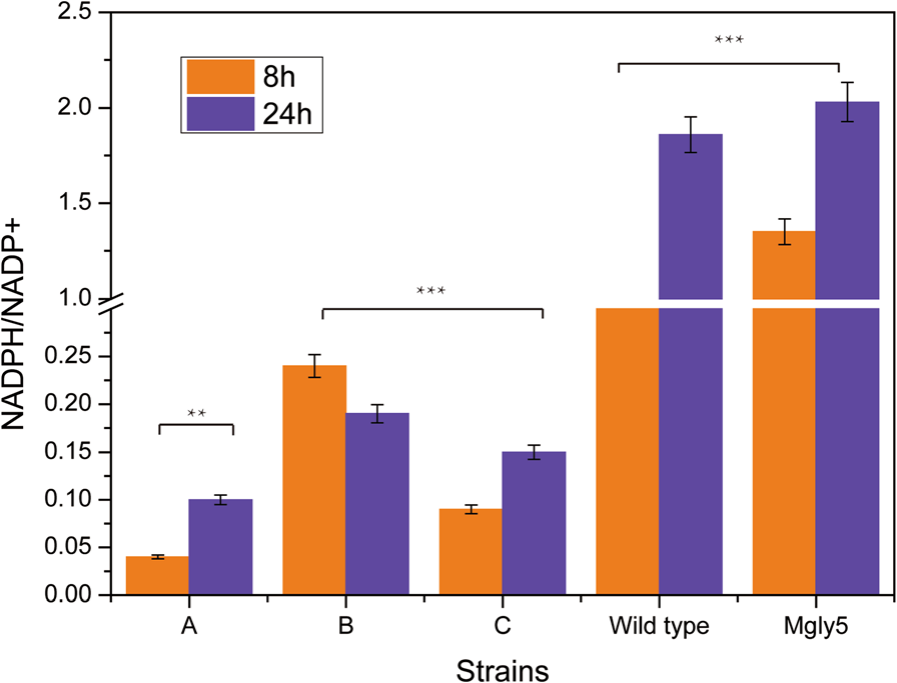
The NADPH/NADP^+^ ratio in different strains. Orange and purple bars represent the ratio after 8 and 24 h of culturing, respectively

The NADPH/NADP^+^ and NADH/NAD^+^ ratios influenced the intracellular redox state and metabolite formation in microorganisms [18]. The production of 1 mol glycolate required 1 mol NADPH and 3 mol NAD^+^ (Additional file 1: Table S1) in ‘C’. Glyoxylate/hydroxypyruvate reductase A (YcdW), which was over-expressed, required NADPH to reduce glyoxylate. Thus, maintaining a balanced NADPH/NADP^+^ pool was extremely important for glycolate synthesis. The NADPH/NADP^+^ ratio in ‘B’ and ‘C’ was decreased significantly compared with the wild-type and plasmid-free strain (Mgly5; Fig. 5). The NADPH/NADP^+^ ratio was highest in ‘B’ (0.24 in 8 h, 0.19 in 24 h) among the three samples, and compared with 0.09 in 8 h and 0.15 in 24 h in ‘C’, and the ratio was lowest in ‘A’ (0.04 in 8 h, 0.1 in 24 h). The transcriptional levels of genes related to NADPH regeneration and consumption are shown in Table 2.

**Table 2:** Gene descriptions related to NADPH

Gene	Reaction stoichiometry	Transcription level (Log_10_(FPKM + 1))		
		A	B	C
*zwf*	Glucose-6-phosphate + NADP^+^ → 6-phospho- D-glucono-1,5-lactone + NADPH + H^+^	0.84	2.08	2.15
*pntA*	NADP^+^ + NADH + H^+^_[periplasm]_ ↔ NADPH + NAD^**+**^_[cytosol]_	1.28	2.15	2.09
*pntB*		1.21	2.12	2.01
*sthA*	NAD^+^ + NADPH → NADH + NADP^+^	1.27	1.73	2.54
*icd*	D-threo-isocitrate + NADP^+^ ↔ 2-oxoglutarate + CO_2_ + NADPH	2.03	3.51	0.88

In *E. coli*, the membrane-bound transhydrogenase (*pntAB*) produced 35–45% of the required NADPH during log phase growth with glucose, while the reactions of the oxidative pentose phosphate pathway and ICDH produced 35–45% and 20–25%, respectively [19]. Thus, deletion of the NADP-dependent ICDH in ‘C’ decreased the regeneration of NADPH and reduced the NADPH/NADP^+^ ratio. Zwf, the first enzyme of the PP pathway, provided a large fraction of the NADPH required for anabolism [20]. Previous research showed that the expression of *zwf* was regulated by growth rate at the transcriptional level [21]. The presence of complex organic carbon and nitrogen sources increased the growth rate of cells under aerobic respiration, hence the expression of *zwf* was up-regulated. Because glycolate production requires NADPH regeneration, we therefore knocked out genes encoding 6-phosphofructokinase I and II (*pfkA* and *pfkB*) to enhance NADPH generation by directing fructose-6-phosphate oxidation through the PP pathway [22, 23]. However, cell growth was severely diminished, and glycolate production was also reduced (data not shown). Thus, the balance between glycolysis and the PP pathway was important for cell growth, and it is not advisable to simultaneously stimulate the PP pathway and weaken glycolysis.

SthA, a soluble, energy-independent transhydrogenase, functioned in NADPH metabolism [19]. It was reported that deletion of *sthA* impaired growth on acetate in *E. coli. SthA* was related to the regulation of intracellular oxidative balance when acetate was used as carbon sources [24]. ‘C’ could use acetate efficiently, implying that the up-regulated *sthA* gene was important in ‘C’ for supporting the self-regulation mechanism corresponding to the imbalance of cofactors.

### Significant changes caused by ICDH knockout

Compared to ‘B’, a strain with native ICDH, there were 68 genes significantly changed in ICDH knockout strain ‘C’ (Additional file 1: Table S2). Among the 68 significantly up- or down-regulated genes, ~ 33 genes were related to N-regulation or amino acid metabolism (Additional file 1: Table S3). Nitrogen assimilation control (NAC) was important for regulating nitrogen metabolism in enteric bacteria, and was closely related to nitrogen regulatory (Ntr) system activated by glutamine starvation [25]. The rut operon in *E. coli* K-12 was discovered as one of the most highly expressed operons under Ntr control and the function of the rut pathway was to release nitrogen [26]. The transcriptional levels of *rutABCDE* in ‘C’ were all up-regulated by 7–15 fold, indicating that ‘C’ utilized more uracil and released more NH_3_ in comparison with ‘B’. NAC activated RNA polymerase σ70 to transcribe operons whose products supplied the cell with ammonium or glutamate from alternative organic sources [27]. The significant up-regulation of NAC in ‘C’ implied a lack of ammonium or glutamate, and some genes were activated to ameliorate the slow growth. Similarly, ammonium assimilation regulatory proteins GlnK and AmtB that were related to the transport of ammonia across biological membranes were significantly up-regulated. GlnK was tightly regulated under nitrogen-rich conditions, yet it was expressed during ammonium depletion and starvation [28]. These results showed that the intracellular glutamate concentration in ‘C’ was lower than in ‘B’, hence glutamate was required in the medium.

**Fig. 6:**
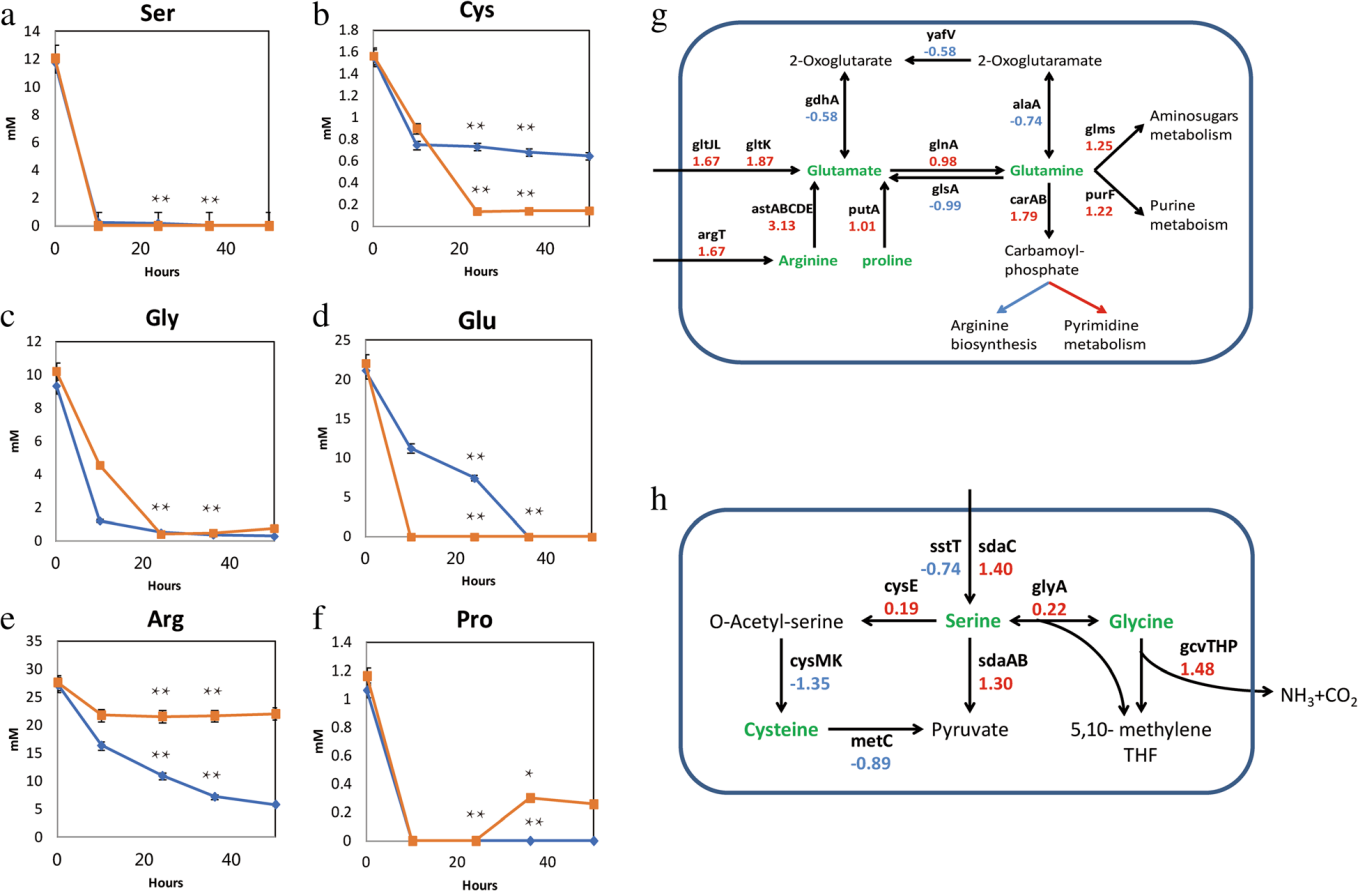
Analysis of amino acid metabolism in the Group B vs. C comparison. **a**–**f** Analysis of amino acids in the fermentation medium of Groups B and C. Blue and orange lines represent the concentrations of amino acids in B and C, respectively. **g**, **h** Glutamate and serine metabolism in *E. coli* K-12 MG1655*.* The name of the enzyme is shown next to the reaction it catalyzes. Values below genes represent the Log_2_FoldChange for the Group B vs. C comparison. Blue and red represent down- and up-regulated genes, respectively. Some pathways include many genes, hence we have only marked genes that changed significantly

We analyzed the free amino acids in the fermentation broth of ‘B’ and ‘C’ at lag phase, mid-log phase, late-log phase, and stationary phase. In the B vs. C comparison, ‘C’ demanded more serine, cysteine, and glutamate (Fig. 6a-f). Glutamate in ‘C’ was completely depleted in mid-log phase (Fig. 6d). It was obvious that the knockout of ICDH increased the demand of glutamine, and the metabolism of arginine and proline was up-regulated to supply the glutamine. (Fig. 6g).

L-serine was a precursor of many other metabolites, including cysteine and glycine, and was completely degraded to pyruvate (Fig. 6h). Expression of the *serABC* cluster in the serine synthesis pathway was not significantly altered in ‘C’. Among the transport system proteins related to serine (*sstT*, *cycA*, *sdaC*, and *tdcC*), *sdaC* was up-regulated, suggesting ‘C’ could use more serine, implying greater conversion via pyruvate metabolism. The glycine cleavage (GcvTHP) enzyme system in *E. coli* catalyzed the oxidative cleavage of glycine to CO_2_ and NH_3_, and the subsequent transfer of a one-carbon methylene unit to tetrahydrofolate, which was used in the biosynthesis of purines, methionine, thymine, and other cellular components [29]. *GcvTHP* and *purNHD* (related to the one-carbon pool via folate) were up-regulated in ‘C’, suggesting that more amino acids including glycine, serine, and glutamine were used for cell growth in ‘C’ than in ‘B’.

**Fig. 7:**
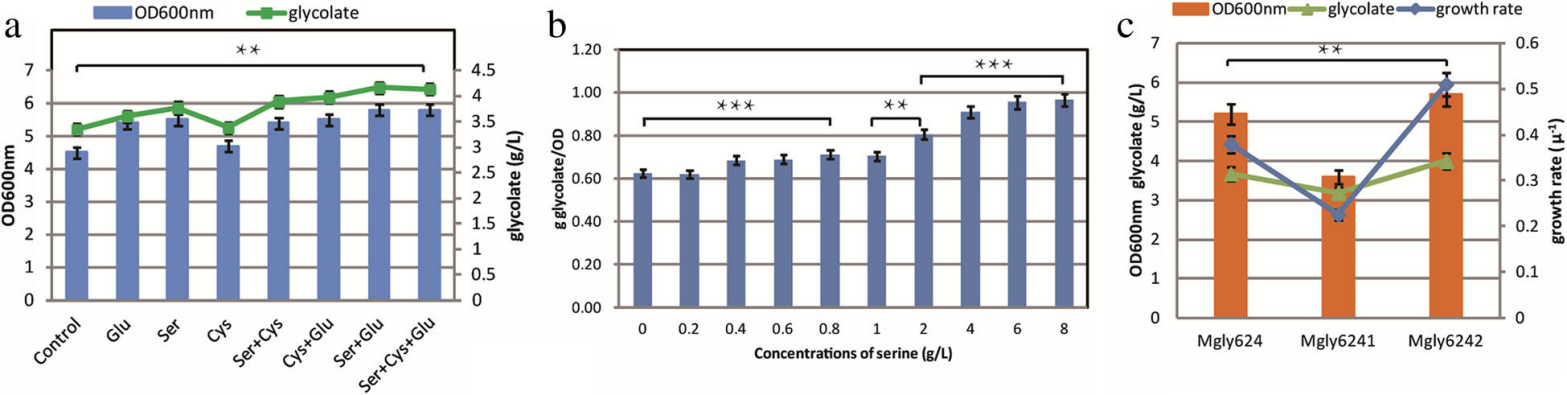
**a** Fermentation characteristics following amino acid addition to Mgly624 cultures. OD_600nm_ (blue bar), and glycolate concentration (green line) are included in the graph. **b** Shake flask fermentation of Mgly624 with additional serine. X-axis represents the different concentrations of additional serine. **c** Fermentation characteristics of *argT* over-expression. OD_600nm_ (orange bar), glycolate concentration (green line), and growth rate (blue line) are included in the graph

To verify the amino acids required in ‘C’, we added extra serine and glutamate to the medium during fermentation, and the effects on biomass and production of glycolate are shown in Fig. 7a. Additional serine and glutamate increased the OD_600nm_ values from 4.5 to 5.8, and the production of glycolate was increased from 3.35 to 4.16 g/L. To further explore the specific role of amino acids, serine was added to the medium of ‘C’. The result showed that the productivity (g glycolate/OD) of glycolate was improved along with the increase of serine, and the titer of glycolate could reach 4.13 g/L with the OD kept stable (Fig. 7b). Based on those results, glutamate was used for the cell growth and serine might be used for the glycolate production. It is known that glutamate could directly make up for the absence of α-ketoglutarate in ICDH knockout strain ‘C’, which was useful for the cell growth. When different concentrations of glutamate were added to the medium, the biomass and glycolate titer increased. OD600nm reached 7.2 with 10 mM glutamate and glycolate titer was increased by 0.4 g/L with 15 mM glutamate. (Additional file 1: Figure S3). However, serine, an unnecessary amino acid, was catalyzed by serine deaminase to produce pyruvate and could directly enter the glyoxylate shunt, which might be the reason for the improvement of glycolate titer.

The arginine succinyltransferase (*astABCDE*, AST), which is necessary for aerobic arginine catabolism, was up-regulated by 8.75-fold in ‘C’. Expression of the enzymes in the AST pathway was regulated by arginine and nitrogen availability [30], arginine degradation was needed during nitrogen-limited growth, and AST also contributed to the degradation of other amino acids [31]. In *E. coli*, arginine catabolism resulted in the assimilation of nitrogen into glutamate and glutamine to provide nitrogen for the synthesis of nitrogen-containing compounds. The biosynthesis of arginine in ‘C’ was decreased, but arginine utilization was increased significantly (Fig. 6g). We speculated that the transcriptional level of the arginine ABC transporter (*argT*) was insufficient for arginine use. We therefore overexpressed the *argT* gene using high- and low-copy number plasmids, and the fermentation results are shown in Fig. 7c. Mgly6242 (harboring plasmid pCOLA-*argT*) exhibited a higher growth rate and biomass than Mgly624, indicating that improving arginine transport could complement the demand for intracellular arginine.

In ‘C’, *sodA* (superoxide dismutase), *fumC* (fumarase C) and *soxS* (superoxide response regulon transcriptional activator), possessing oxidative stress response capabilities, were up-regulated by 5.43-, 3.29- and 4-fold respectively. We speculated that the changes were caused by the excessive superoxide radical. NADP^+^-dependent ICDH, a source of NADPH for the regeneration of cellular antioxidants, is important for oxidative damage protection [32]. However, in ICDH-deleted strain ‘C’, the cellular antioxidant defense was impaired, thus those genes were up-regulated for maintaining the intercellular oxidation-reduction balance [33–35].

**Fig. 8:**
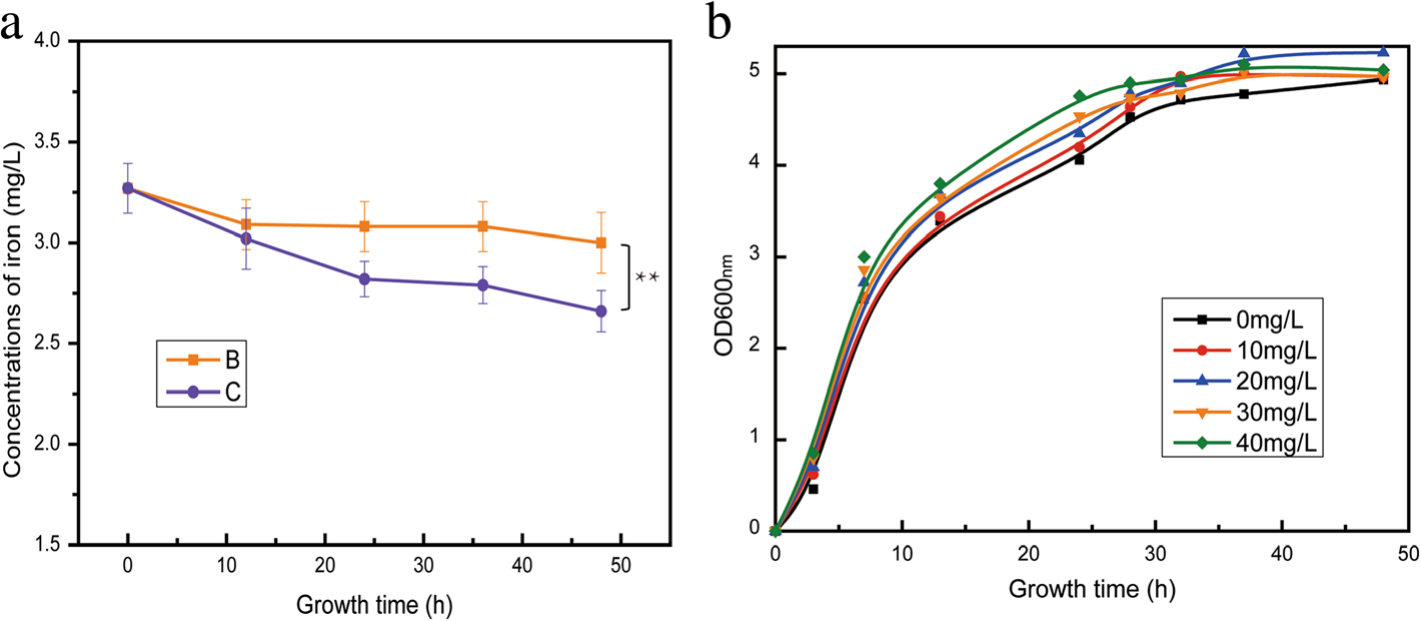
**a** Iron concentrations in the fermentation medium of Groups B and C during the fermentation process. **b** Growth curve of Mgly624 with different concentrations of Fe^2+^ addition

Meanwhile, it was noted that *fecABCE* (ferric citrate ABC transporter) genes were significantly up-regulated in ‘C’. The transcription of ferric citrate transport system was activated by intercellular iron limitation [36], and the up-regulation of *fecABCE* caused iron starvation in ‘C’. Iron limitation resulted in decreased activities of many enzymes and enzyme systems associated with the respiratory chain during the growth of *E. coli*, such as NADH-nitrate reductase, NADH-cytochrome c reductase and succinate dehydrogenase, and resulted in a loss of energy-coupling since many components in the respiratory chain are iron-containing proteins, such as NADH dehydrogenase, flavin protein and cytochrome [37]. In addition, iron-sulfur (Fe-S) cluster, an important component of the respiratory chain, was required for the regeneration of many reductase [38], and it was noted that the sulfur metabolism was increased in ‘C’ (Fig. 3b), which was consistent with the up-regulation of *fecABCE*. To verify the iron shortage in ‘C’, the iron concentrations were measured in the medium of ‘B’ and ‘C’ respectively (Fig. 8a). More iron was consumed in the medium of ‘C’ during the fermentation process. Especially, when the Fe^2+^ was supplemented, the growth rate of Mgly624 was accelerated, and the time taken to reach the stable period was shortened (Fig. 8b), while the titer of glycolate was not increased. However, owing to the less reducing equivalent, Mgly624 did not have the same phenotype when the Fe^3+^ was added to the medium (Additional file 1: Figure S4). In summary, when ICDH was deleted, the addition of Fe^2+^ could compensate for the lack of reducing equivalents caused by NADPH decrease, indicating that the Fe^2+^ transport was essential for the ICDH knockout strain.

## Discussion

Glycolate is produced by cleaving isocitrate to glyoxylic acid through the glycolysis, and glyoxylic acid is reduced to glycolate. The glycolysis can be up-regulated under conditions of oxidative stress, host infection, antibiotic stress, and other stresses. This implies that the glycolysis could be triggered by many extreme environmental stimuli [39]. M9 salt medium was reported to be suitable for the production of glycolate by the engineered strains because a limited medium was best for bypassing the glycolysis. We constructed engineered strain Mgly534 to produce glycolate [5], but the growth in M9 was poor. However, when organic nitrogen sources were added to improve cell growth, it was accompanied by a decreased glycolate production. Thus, the balance between the TCA cycle and the glycolysis is a key factor for the production of glycolate and biomass. In the previous work, we weakened the TCA cycle by overexpressing isocitrate dehydrogenase kinase/phosphatase (*aceK*), but the activity of ICDH was not well suppressed. In the present study, we deleted ICDH to block carbon flow to the TCA cycle, and the ICDH-deletion strain (Mgly624) displayed differences in growth and fermentation characteristics compared with the control strain. Glycolate production was not reduced with the increase in organic nitrogen sources, which achieved the balance between glycolate production and cell growth.

In contrast to Mgly534, the glycolate production in Mgly624 did not increase significantly. However, Mgly624 possessed considerable advantages in fermentation process. Compared with M9 salt medium, with the characteristic of enhanced glyoxylate shunt and weakened TCA for Mgly534, the growth of Mgly534 was improved on the rich medium, while the glycolate decreased and acetate accumulated. Currently, the reported glycolate production reached a very high level, thus it is essential to maintain the stability and productivity during fermentation process. Though the less significant change in glycolate production, the knockout of ICDH solved this problem perfectly, and the transcriptome was performed to reveal the mechanism why the growth of Mgly624 did not compete with glycolate production on the rich medium.

Among the 68 significantly changed genes, there were some related to the transport of other carbon sources. *LamB* and *malEFM*, as the maltose transporter, were significantly up-regulated in ICDH-deletion strain, indicating that there were some other carbon complex in the medium except the carbon in amino acids, and more carbon sources were needed for ICDH-deletion strain. When the organic nitrogen source was present, the yield of glycolate in Mgly624 was higher than the theoretical yield (0.86 g/g-glucose) during fermentation process, which was due to the carbon sources in the yeast extract and tryptone. Though the yield could not be accurately calculated in this medium, Mgly624 still exhibited excellent glycolate production capabilities compared with Mgly534 and these results provide a better understanding of the Mgly624 and glycolate fermentation optimization.

Based on transcriptome data, our present findings improved the balance between the cell growth and glycolate production. At present, there are some studies where researchers carry out RNA-Seq-based flux balance analysis modeling [40]. Those in silico methods could provide systemic analysis of metabolic balance, which would be helpful for the further study of glycolate production.

## Conclusions

Our results indicated that Mgly624 achieved a better balance between glycolate production and biomass with addition of organic nitrogen sources. NADPH is a key cofactor for the production of glycolate, and organic nitrogen sources slightly increases NADPH availability. Through the analysis of significantly changed genes, the culture medium was optimized and arginine transporter gene was overexpressed in Mgly624, the production of glycolate was increased to 4.16 g/L in shake flasks. Our work here provide a better understanding of the ICDH knockout on cellular physiology and glycolate production.

## Methods

### Culture conditions

The strains used in this study are listed in Table 1. In batch fermentation, 3 L of M9 salt medium with 8 g/L tryptone, 2 g/L yeast extract, and 10 g/L glucose was used in a 5 L bioreactor, and 2 M HCl and 2 M NaOH were used to maintain the pH around 7.0. DO was controlled at 50–60%. A 100 mL cell culture was grown overnight in Luria Broth (LB) medium at 37 °C then transferred to the bioreactor. After the OD_600nm_ reached 1.0, 0.1 mM IPTG was added to induce transcription, then culturing was continued at 30 °C.

To identify the optimal concentrations of organic nitrogen sources, 1, 2, 3, 4, and 5 g/L yeast extract was tested along with 2, 4, 6, 8, and 10 g/L tryptone in the M9 salt medium (containing 8 g/L glucose). To study the effects of amino acids on fermentation, serine (10 mM), glutamate (20 mM), or cysteine (5 mM) was added to the M9 salt medium (with 8 g/L tryptone, 2 g/L yeast extract, and 8 g/L glucose), as well as combinations of amino acids. The medium was neutralized with NaOH. To study the effects of Fe^2+^ on cell growth, 50, 100, 150, 200 mg/L FeSO_4_∙7H_2_O was added to the medium.

Strains were cultured in LB overnight at 37 °C with shaking at 250 rpm, and 1 mL of cell culture was transferred to a 250 mL shake flask and cultured at 37 °C with shaking at 250 rpm. When the OD_600nm_ reached 0.8, cultures were induced with 0.1 mM IPTG and cultured at 30 °C with shaking at 250 rpm. Samples were taken every 4 h and OD_600nm_ values and metabolite concentrations were measured.

### Plasmid construction

Three plasmids were constructed for the production of glycolate (Table 1). The primers used in this study are listed in Table 3. For the construction of pTrc99A-*aceA*-*ycdW*, the glyoxylate reductase gene (*ycdW*) and isocitrate lyase (*aceA*) were amplified from pTrc99A-*aceAK-ycdW* [5] using primers pAY-F (*Eco*RΙ) and pAY-R (*Bam*HΙ), and the resulting product was ligated into plasmid pTrc99A, which was digested with *Eco*RΙ and *Bam*HΙ. For construction of pRSF-*argT* and pCOLA-*argT*, the *argT* gene was amplified from *E. coli* genomic DNA using primers p*argT*-F (*Nco*I) and p*argT*-R (*Bam*HΙ), and the product was ligated into plasmids pRSFDuet-1 and pCOLADuet-1, respectively, which were digested with *Nco*I and *Bam*HΙ*.*

**Table 3:** List of DNA oligo nucleotide primers used in the cloning of genes

Primer name	Sequence(5′-3′)
pAY-F	CGAATTCCAACGCTTTTCGGGAGTCAGTAT
pAY-R	CGGGATCCTTAGAACTGCGATTCTTCAGTGG
p*argT*-F	CATGCCATGGATGAAGAAGTCGATTCTCGCTCT
p*argT*-R	CGGGATCCTCAGTCACCGTAGACATTAAAGT
p*icd*-F	GGCAGACGAGCAAACCAGTAGCGCTCGAAGGAGAGGTGAGTGTAGGCTGGAGCTGCTTC
p*icd*-R	GCGTTACGCTCCCGTTAATAAATTTAACAAACTACGGCAATGGGAATTAGCCATGGTCC
veri-*icd*-F	AACATCGTAGGGTTTATTGAACAGGA
veri-*icd*-R	GAGCGGGTGTAAGGAGTGGTAAT

“____” indicates the cleavage site of restriction endonuclease

### Strain engineering

The glycolate engineering strain was *E. coli* K-12 MG1655 (DE3). For strain engineering, LB medium was used, and λ-red recombination [41] was used for the deletion of *glcB*, *aceB*, *aldA*, *ldhA*, and *glcDEF*, resulting in strain Mgly5. Using Mgly5, ICDH was knocked out to generate Mgly6. A deletion cassette (containing the kanamycin resistance gene with an FRT site and a 39 bp homologous region) was amplified from pKD4 using primers p*icd*-F and p*icd*-R. Plasmid pKD46 was transferred into Mgly5 and cells were induced with 10 mM arabinose to express the λ-red recombinase. The deletion cassette was transferred to Mgly5 with pKD46. To obtain colonies in which the *icd* gene was replaced by the kan-cassette, colony PCR was performed using primers veri-*icd*-F and veri-*icd*-R. Plasmid pCP20 was transferred into the right colony and the knock-out cassette was eliminated by Flp recombinase following expression at 37 °C for 12 h. The resulting mutant strain Mgly6 was identified by colony PCR with primers veri-*icd*-F and veri-*icd*-R, and pCP20 was eliminated at high temperature (37 °C).

### Metabolite analysis

To measure the concentrations of metabolites in the broth, the cell culture was centrifuged at 12,000 g for 5 min, and the supernatant was filtered through a 0.22 μM syringe filter. The concentration of glucose, glycolate and acetate was analyzed by HPLC (HITACHI, Japan) equipped with an Aminex HPX-87H column (Bio-Rad, Hercules, CA, USA) at 55 °C using a refractive index detector with a mobile phase of 5 mM sulfuric acid.

For analyzing free amino acids in the fermentation broth, an automatic Amino Acid Analyzer (HITACHI L-8900, Japan) was used [42]. The cell culture was centrifuged at 12,000 g for 5 min, and 10% sulfosalicylic was added to the supernatant in a 1:1 ratio. The mixture as incubated at 4 °C for more than 4 h, centrifuged at 12,000 g for 10 min, and 0.02 M HCl was added to the appropriate concentration and filtered through a 0.22 μM syringe filter for amino acid analysis.

The analysis of Fe was performed on Atomic absorption spectroscopy analyzer (Varian AA240FS-GTA120, USA). The cell culture was centrifuged at 12,000 g for 10 min, and 1 mol/L HNO_3_ was added to the appropriate concentration.

### NADPH and NADP^+^ assays

Cells were cultured overnight in LB medium at 37 °C and 1 mL of cell culture was transferred to 50 mL of fresh fermentation medium in a 250 mL shake flask and cultured at 37 °C with 250 rpm. When the OD_600nm_ reached 0.8, cultures were induced with 0.1 mM IPTG and cells were harvested at 8 h and 24 h. Cell cultures were divided into two separate centrifuge tubes (1 mL each) and centrifuged at 4000 g for 2 min at 4 °C, and the supernatant was removed. To isolate the oxidized form (NADP^+^), 0.3 mL of 0.4 M HCl was added to resuspend the cell pellet. To isolate the reduced form (NADPH), 0.3 mL of 0.4 M NaOH was added to resuspend the cell pellet. All samples were heated to 55 °C for 10 min, immediately placed on ice, and neutralized (0.3 mL 0.4 M NaOH for the oxidized form; 0.3 mL 0.4 M HCl for the reduced form). Samples were centrifuged (4 °C, 10 min, 12,000 g) and supernatants were transferred to new centrifuge tubes for cofactor assays.

Reaction mixtures contained 3× water, 1× 1.0 M TRICINE-NaOH (pH 8.0), 1× 40 mM EDTA, 1 × 25 mM G6P, 1× 4.2 mM Thiazolyl Blue (MTT), and 2× 16.6 mM phenazine ethosulfate. A 90 μL reaction mixture was aliquoted into individual wells of a 96-well plate, and 5 μL of standards or samples were added to each well. The plate was heated to 30 °C, and enzymatic reactions were initiated by the addition of 5 μL G6P dehydrogenase (14 U/mL in 0.1 M BICINE, pH 8.0). The plate was incubated at 30 °C with continuous shaking, and the absorbance at 570 nm, which was the spectral peak of reduced MTT that was increased during the reaction, was read every 30–60 s. Slopes arising from plots of absorbance at 570 nm over time were generated for NADPH and NADP^+^ standards as well as all samples. Standard curves based on the correlation between standard concentration and slope were used to calculate the absolute concentrations, and values were normalized against the ratio with the OD_600nm_ value of the original cell culture sample [43].

### Transcriptome analysis by RNA-Seq

Cells for RNA-Seq were cultured in a 5 L bioreactor with 3 L fermentation medium. After batch fermentation, 50 mL cells were harvested at mid-log phase, flash-frozen in liquid nitrogen, and stored at − 80 °C. Total RNA isolation and transcriptome sequencing were performed by GENEWIZ (Suzhou, China) according to a standard procedure. RNA samples were extracted and quality was assessed by an Agilent Bioanalyzer 2100 and agarose gel electrophoresis. After removing ribosomal RNA, libraries for strand-specific RNA sequencing were constructed with a NEBNext Ultra Directional RNA Library Prep Kit for Illumina. An ABI 7500 real-time PCR system KAPA SYBR green fast universal 2× qPCR master mix was used for library quantification. A TruSeq PE Cluster Kit V4 was used for cBOT automatic clustering. The library was sequenced on an Illumina HiSeq platform with a TruSeq SBS Kit v4-HS. After obtaining raw sequencing data, FastQC (V0.10.1) [44] was used to evaluate sequencing data quality by calculating the sequencing error rate (e) and base weight value (Q_phred_) [44].

Filtered clean data were mapped to the reference genome (*Escherichia coli str. K-12 substr. MG1655*) by bowtie2 (V2.1.9) [45]. Rockhopper software was used to analyze the sequencing data to identify novel transcript regions [46]. Gene expression calculations were performed with Htseq software (V0.6.1) [47] which uses the FPKM method to calculate gene expression levels. In order to calculate DEGs, the input data was the read count data obtained in the analysis of gene expression levels. Genetic diversity analysis was performed using Bioconductor software package DESeq2 (V1.6.3) [48].

GO [49] and KEGG [50] enrichment analyses were performed by GENEWIZ (Suzhou, China).

### Statistical analysis

All data in this study were stated as means ± standard error of mean (SEM), and analysis by Student’s *t*-test, with *p < 0.05, **p < 0.01 and ***p < 0.001, ns, no significant.

## Additional files

Additional file 1: **Figure S1.** Shake flask fermentation of Mgly624 in different concentrations of organic nitrogen source. **Figure S2.** GO histogram of DEGs. **Figure S3.** Shake flask fermentation of Mgly624 in different concentrations of glutamate. **Figure S4.** Growth curve following Fe^3+^ addition to Mgly624 cultures. **Table S1.** The stoichiometry of producing glycolate from glucose in Mgly624. **Table S2.** Significantly altered genes in the Group B vs. C comparison. **Table S3**. Description of significantly altered genes related to N-regulation and amino acids metabolism in the Group B vs. C comparison. (DOCX 207 kb)

Additional file 2: All FPKM values of all genes. (XLSX 345 kb)

## Abbreviations

DEGs: Differential expression of genes
DO: Dissolved oxygen
EMP: Glycolysis pathway
GO: Gene Ontology
GS: Glyoxylate shunt
ICDH: Isocitrate dehydrogenase
IPTG: Isopropyl β-D-1-Thiogalactopyranoside
KEGG: Kyoto Encyclopedia of Genes and Genomes
LB: Luria Broth
NAC: Nitrogen assimilation control
OAA: Oxaloacetic acid
OD: Optical density
PP: Pentose phosphate
TCA: Tricarboxylic acid
