# Supplementary material for: Systematic analysis of the effects of different nitrogen source and ICDH knockout on glycolate synthesis in Escherichia coli
Source: J Biol Eng. 2019 Apr 4;13:30. doi: 10.1186/s13036-019-0159-2 (PMC6449901; doi:10.1186/s13036-019-0159-2)
Supplement: Supplementary file 1 — Figure S1. Shake flask fermentation of Mgly624 in different concentrations of organic nitrogen source. Figure S2. GO histogram of DEGs. Figure S3. Shake flask fermentation of Mgly624 in different concentrations of glutamate. Figure S4. Growth curve following Fe3+ addition to Mgly624 cultures. Table S1. The stoichiometry of producing glycolate from glucose in Mgly624. Table S2. Significantly altered genes in the Group B vs. C comparison. Table S3. Description of significantly altered genes related to N-regulation and amino acids metabolism in the Group B vs. C comparison. (DOCX 207 kb) [file 13036_2019_159_MOESM1_ESM.docx]

**Systematic analysis of the effects of different nitrogen source and ICDH knockout on glycolate synthesis in *Escherichia coli***

Kangjia Zhu^1,2,3#^, Guohui Li^1,2,3#^, Ren Wei^4^, Yin Mao^1,2,3^, Yunying Zhao^1,2,3^, Aiyong He^5^, Zhonghu Bai^1,2,3^ and Yu Deng^1,2,3,5*^

^1^ National Engineering Laboratory for Cereal Fermentation Technology (NELCF), Jiangnan University, 1800 Lihu Road, Wuxi, Jiangsu 214122, China.

^2^ School of Biotechnology, Jiangnan University, 1800 Lihu Rd, Wuxi, Jiangsu 214122, China.

^3^Jiangsu Provincial Research Center for Bioactive Product Processing Technology, Jiangnan University

^4^Institute of Biochemistry, Leipzig University, Johannisallee 23, D-04103, Leipzig, Germany

^5^Jiangsu Key Laboratory for Biomass-based Energy and Enzyme Technology, Huaiyin Normal University, Huaian 223300, China

# These people contributed equally to this work.

^*^ Correspondence to:

Yu Deng: National Engineering Laboratory for Cereal Fermentation Technology (NELCF), Jiangnan University, 1800 Lihu Road, Wuxi, Jiangsu 214122, China.

Phone: +86-510-85329031, Fax: +86-510-85918312

E-mail: dengyu@jiangnan.edu.cn

**(a)**

**(b)**

**Supplementary Figure S1.** **Shake flask fermentation of Mgly624 in different concentrations of organic nitrogen source**. (**a**) Shake flask fermentation of Mgly624 in different concentration of yeast extract. (**b**) Shake flask fermentation of Mgly624 in different concentrations of tryptone.

**(a)**


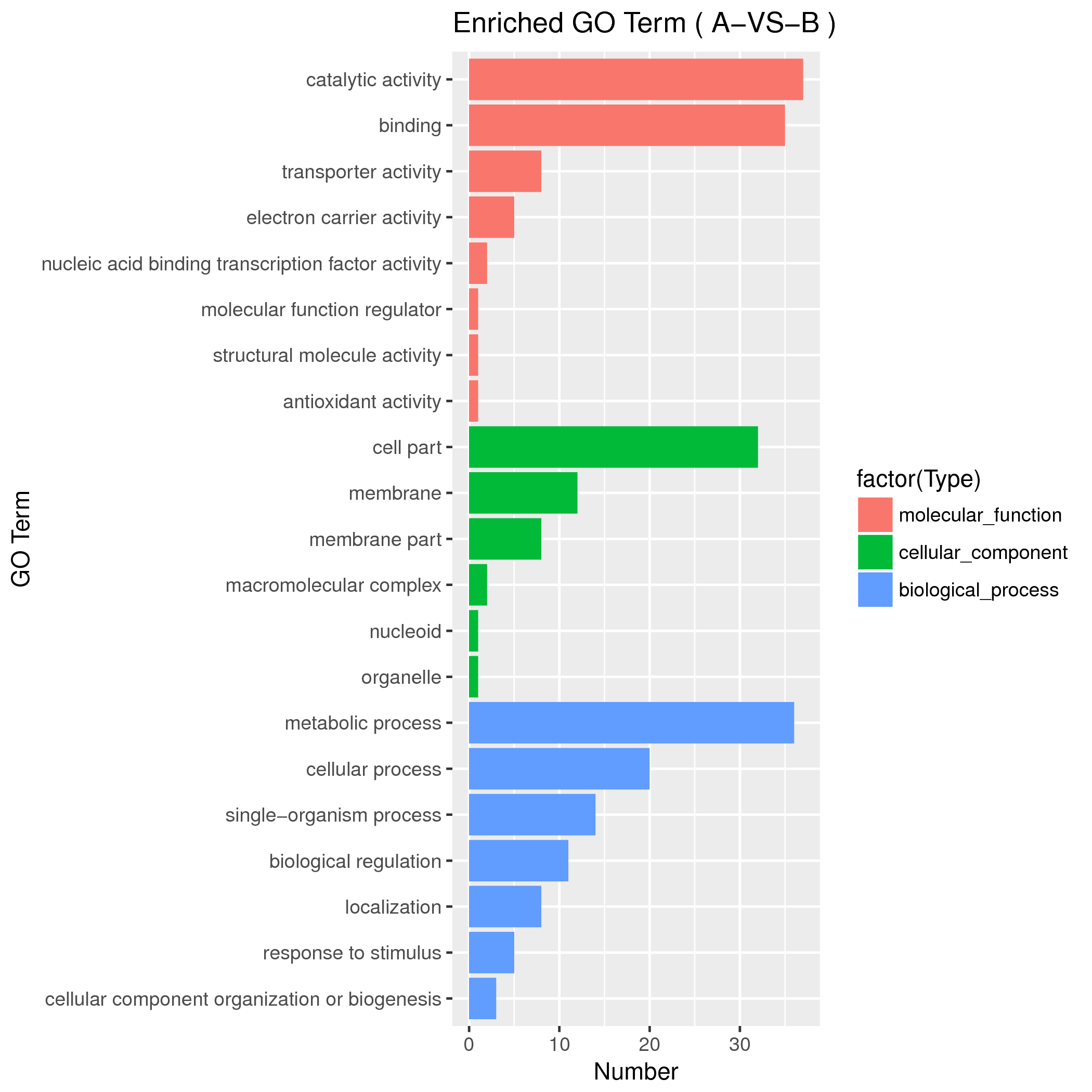


**(b)**


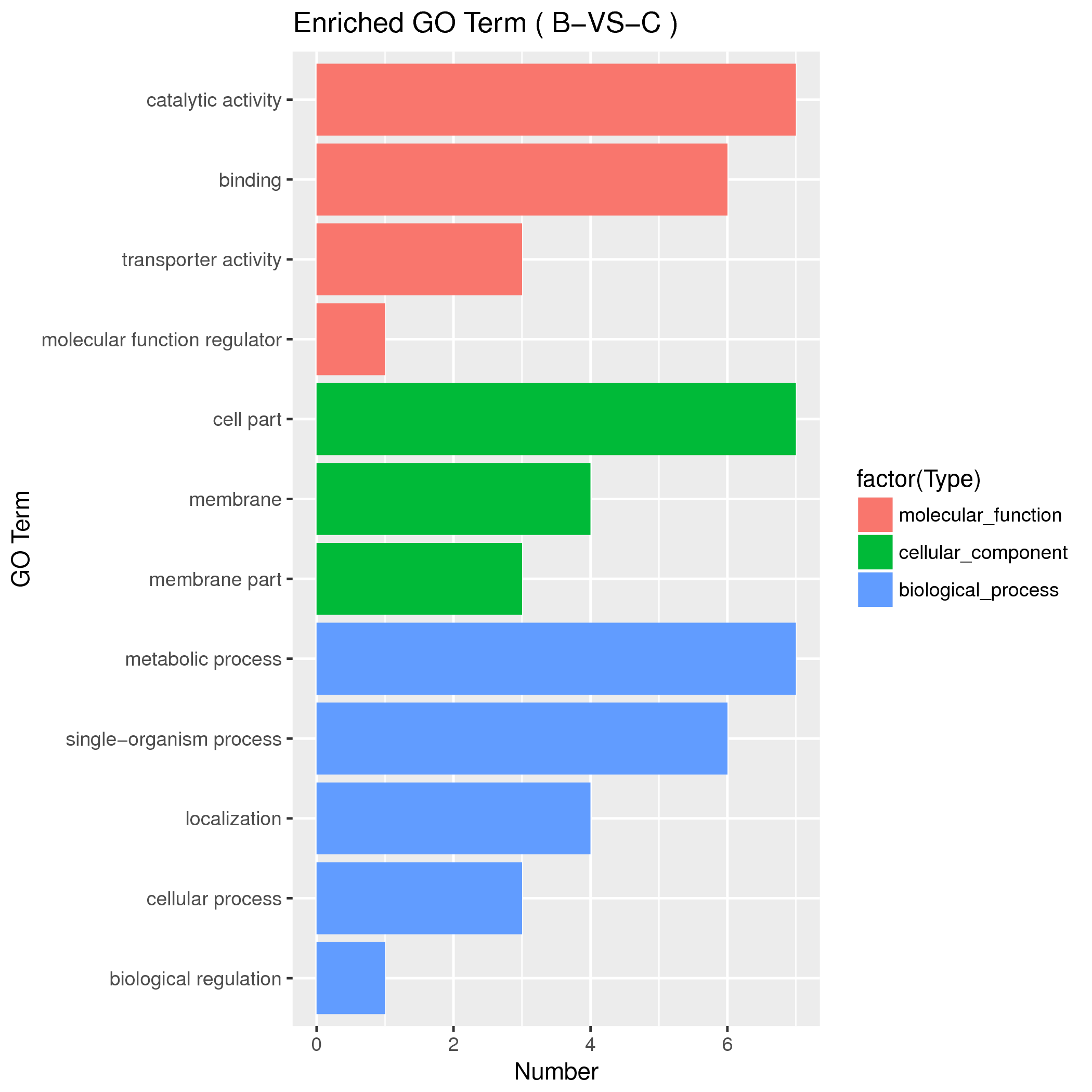


**Supplementary Figure S2. GO histogram of DEGs**. The GO term on the ordinate is the number of DEGs in the term. Different colors are used to distinguish biological processes, cellular components and molecular functions. (**a**) Group A-vs-B; (**b**) Group B-vs-C

**Supplementary Figure S3.** Shake flask fermentation of Mgly624 in different concentrations of glutamate

**Supplementary Figure S4.** Growth curve following Fe^3+^ addition to Mgly624 cultures.

**TABLE S1** The stoichiometry of producing glycolate from glucose in Mgly624

| **1:** Glu+2ADP+2Pi+2NAD^+^ = 2PYR+2NADH+2H^+^+2H_2_O+2ATP |
| --- |
| **2:** PYR+CoA+NAD^+^=acetyl-CoA+CO_2_+NADH |
| **3:** acetyl-CoA+OAA+H_2_O=ICI+CoA+2H^+^ |
| **4:** ICI=Glyoxylate+SUC |
| **5:** Glyoxylate+NADPH+H^+^=Glycolate+NADP^+^ |
| **6:** SUC+FAD+NAD^+^=OAA+FADH_2_+NADH |
| Glu+2ADP+2Pi+6NAD^+^+2NADPH+2FAD=2Glycolate+6NADH+2NADP^+^+2H^+^+2CO_2_+2FADH_2_+2ATP |

**TABLE S2** Significantly altered genes in the Group B vs. C comparison

| GeneName | log2FoldChange |
| --- | --- |
| icd | -5.52 |
| yohK | -2.44 |
| yjiY | -2.35 |
| yohJ | -2.33 |
| aceK | -2.33 |
| adiY | -2.15 |
| cysA | -2.06 |
| bhsA | -2.04 |
| yjiG | -2.01 |
| thrA | -1.89 |
| yeeD | -1.89 |
| asnA | -1.87 |
| ycgI | -1.83 |
| hokD | -1.83 |
| yjiH | -1.83 |
| yeaR | -1.80 |
| relE | -1.57 |
| leuB | -1.56 |
| cysC | -1.47 |
| fadA | 1.48 |
| hisP | 1.51 |
| sdhA | 1.64 |
| gltJ | 1.64 |
| gltL | 1.66 |
| cpxP | 1.67 |
| argT | 1.67 |
| codB | 1.68 |
| fumC | 1.72 |
| hisM | 1.74 |
| carA | 1.79 |
| gltK | 1.87 |
| soxS | 2.00 |
| malM | 2.03 |
| fecE | 2.13 |
| yhfZ | 2.15 |
| aspA | 2.19 |
| malF | 2.23 |
| fecC | 2.30 |
| glxR | 2.32 |
| hyi | 2.34 |
| fecB | 2.38 |
| sodA | 2.44 |
| malE | 2.45 |
| malK | 2.49 |
| lamB | 2.53 |
| ddpF | 2.54 |
| fecA | 2.55 |
| astC | 2.60 |
| ddpC | 2.73 |
| acs | 2.75 |
| ddpD | 2.88 |
| rutD | 2.89 |
| astB | 3.03 |
| rutE | 3.08 |
| astE | 3.13 |
| astA | 3.13 |
| rutC | 3.16 |
| astD | 3.25 |
| yhdW | 3.27 |
| amtB | 3.65 |
| rutB | 3.73 |
| ddpB | 3.77 |
| rutA | 3.92 |
| nac | 4.00 |
| ddpA | 4.12 |
| ddpX | 4.26 |
| yedL | 4.26 |
| glnK | 4.59 |

**TABLE S3** Description of significantly altered genes related to N-regulation and amino acids metabolism in the Group B vs. C comparison

| **Gene** | **Description** | **Log2FC (FDR < 0.05)** |
| --- | --- | --- |
| *hisP* | Histidine transporter | 1.51 |
| *hisM* |  | 1.74 |
| *carA* | Glutamine amidotransferase | 1.79 |
| *gltJ* | Glutamate transport | 1.64 |
| *gltL* |  | 1.66 |
| *gltK* |  | 1.87 |
| *argT* | Lysine/arginine/ornithine transporter subunit | 1.67 |
| *codB* | Cytosine transporter | 1.68 |
| *aspA* | aspartate ammonia-lyase | 2.19 |
| *astC* | Arginine catabolism | 2.6 |
| *astB* |  | 3.03 |
| *astE* |  | 3.13 |
| *astA* |  | 3.13 |
| *astD* |  | 3.23 |
| *yhdW* | Polar amino acid transport | 3.27 |
| *amtB* | ammonium transporter | 3.65 |
| *ddpF* | D-ala-D-ala dipeptide transport and dipeptidase | 2.54 |
| *ddpC* |  | 2.73 |
| *ddpD* |  | 2.88 |
| *ddpB* |  | 3.77 |
| *ddpA* |  | 4.12 |
| *ddpX* |  | 4.26 |
| *rutD* | Pyrimidine utilization pathway | 2.89 |
| *rutE* |  | 3.08 |
| *rutC* |  | 3.16 |
| *rutB* |  | 3.73 |
| *rutA* |  | 3.92 |
| *nac* | N-regulation | 4.00 |
| *glnK* | Nitrogen assimilation regulatory protein | 4.59 |
| *cysC* | Cysteine synthesis | −1.47 |
| *cysA* |  | −2.06 |
| *asnA* | Asparagine synthetase A | −1.87 |
| *adiY* | Arginine degradation regulator | −2.15 |
